# Acquired lymphatic malformations of the buccal mucosa: A case report

**DOI:** 10.1002/ccr3.1756

**Published:** 2018-08-15

**Authors:** Atsushi Abe, Kenichi Kurita, Yu Ito

**Affiliations:** ^1^ Department of Oral and Maxillofacial Surgery Nagoya Ekisaikai Hospital Nagoya Japan; ^2^ Department of Oral and Maxillofacial Surgery Aichi‐gakuin University Nagoya Japan

**Keywords:** buccal mucosa, lymphatic malformation, trauma

## Abstract

Lymphatic malformation is a rare disorder caused by expansion and proliferation of the lymphatic vessels. About 50% of lymphatic malformations are present at birth, and 90% are diagnosed before the age of 2 years. The most common site of lymphatic malformation in the oral cavity is the anterior two‐thirds of the tongue, and involvement of the buccal mucosa is relatively unusual. Treatment of lymphatic malformation includes surgical excision, sclerotherapy, laser therapy, radiofrequency ablation, or a combination of these interventions. In many cases, surgery or sclerotherapy is the treatment of choice. However, there is currently no consensus on an optimal treatment approach. Here, we report an unusual case of lymphatic malformation in the buccal mucosa of an older adult that was surgically excised.

## 1 INTRODUCTION

About 75% of all lymphatic malformations occur in the head and neck area.^1^ The most common site of an oral lymphatic malformation is the anterior two‐thirds of the tongue.^2^ However, lymphatic malformations can occur at various sites in the oral cavity. Congenital lymphatic malformations are present at birth, and about 90% of cases are diagnosed before the age of 2 years.^3^ A diagnosis of acquired lymphatic malformations is exceptionally rare in an elderly person. Here, we report a case of acquired lymphatic malformation in the left buccal mucosa in an elderly patient that was surgically excised.

## 2 CASE REPORT

**Figure 1:**
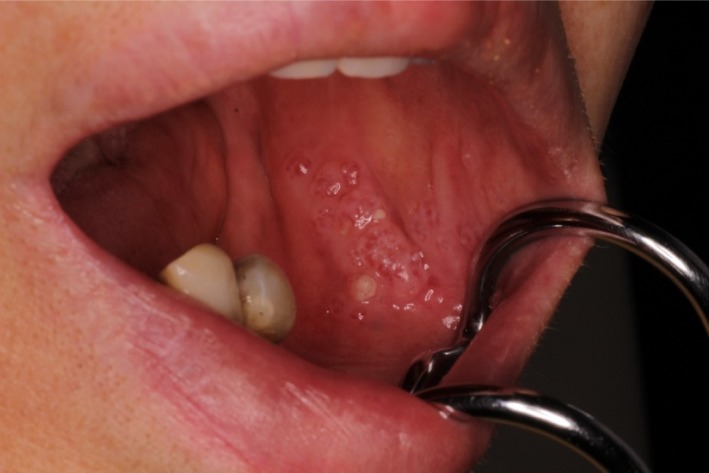
Diffuse swelling of the left buccal mucosa, measuring about 15 × 30 mm

**Figure 2:**
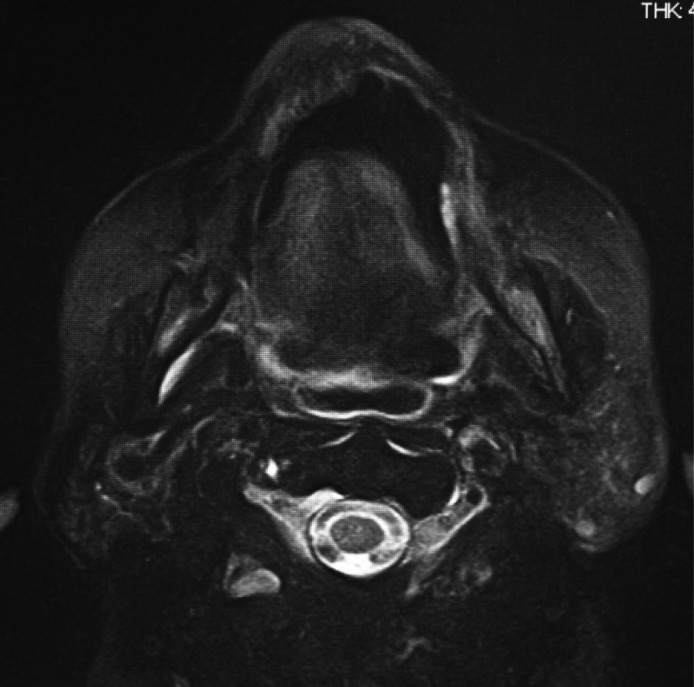
A T2‐weighted magnetic resonance image showing a lymphatic malformation with a clearly distinguishable outline of the buccinator muscles

**Figure 3:**
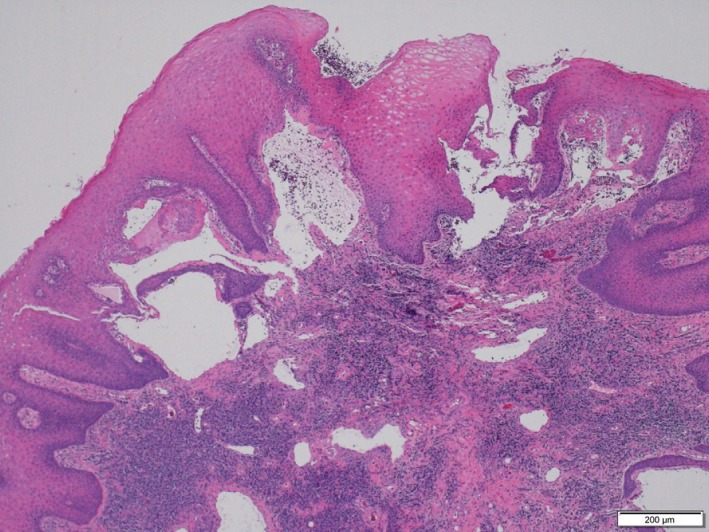
The subcuticular lesion is covered by stratified squamous epithelium with an expanded lumen

**Figure 4:**
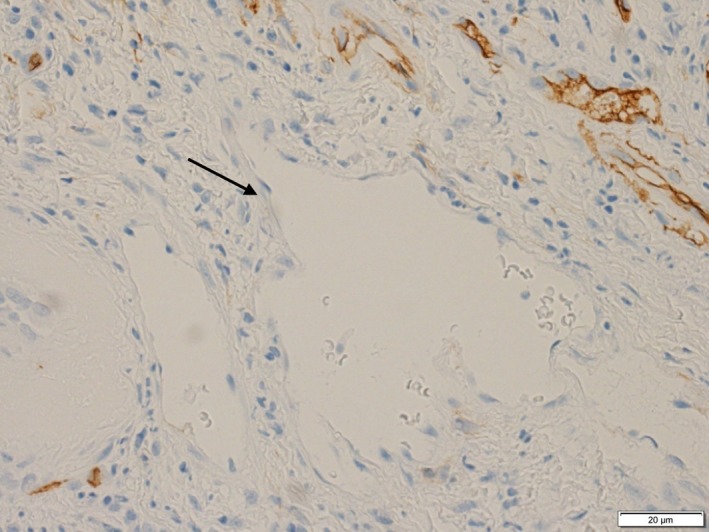
The vasculature was CD34‐negative, suggesting the presence of a blood vessel (arrow)

**Figure 5:**
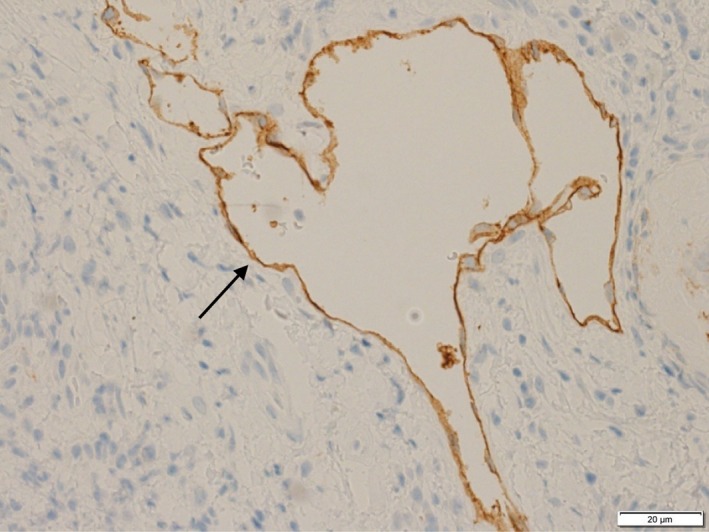
The vasculature was D2‐40 (podoplanin)‐positive, suggesting the presence of a lymph duct (arrow)

A 68‐year‐old woman presented to the Department of Oral and Maxillofacial Surgery at Nagoya Ekisai Hospital (Nagoya, Japan) with a chief complaint of malaise and a 7‐month history of swelling of the left buccal mucosa. The patient had no congenital swelling of the left buccal mucosa at birth and no history of systemic disease or relevant family history. The patient had undergone maxillary molar restoration treatment 2 years earlier, after which she reported biting regularly on her buccal mucosa. On most occasions, the wound had healed within a week, so she had not sought medical treatment. An extraoral examination revealed no facial swelling or asymmetry. However, an intraoral examination revealed an area of diffuse swelling on the left buccal mucosa measuring about 15 mm × 30 mm and containing a papillary lesion with multiple red, blue, and clear pebble‐like vesicles (Figure 1). On palpation, the lesion was nontender and soft. The swelling had not expanded to the veins and was pulsatile. An orthopantomogram confirmed that the adjacent bone was intact. Magnetic resonance imaging revealed a soft tissue mass with a clearly distinguishable outline of the buccinator muscle (Figure 2). The lesion was surgically excised under local anesthesia with a margin of 3 mm and a depth of 2 mm via the inside surface of the fascia of the buccinator muscle. The outcome was favorable. Pathologic examination of the specimen revealed expanded lymphatic vessels lined by thin endothelial cells and containing lymphatic fluid. A diagnosis of lymphatic malformation was confirmed on histopathology and immunohistochemical studies. Immunohistochemistry was negative for vascular markers such as CD31 and CD34, and the lymphatics stained specifically for D2‐40 (podoplanin) (Figures 3, 4, 5). On follow‐up, the wound was found to have healed with no evidence of trismus or recurrence. The patient continued to be recurrence‐free at her 2‐year follow‐up. Informed consent was obtained from the patient, and the procedures were in accordance with the Helsinki Declaration.

## 3 DISCUSSION

Lymphatic malformation is caused by failure of the lymphatic communication, lymphatic obstruction, fluid retention and cell proliferation. Although lymphatic malformation can occur at any age, cases in adults are very rare.^1^ The morbidity rate associated with lymphatic malformation is reportedly 1.2%‐2.8%.^2^ A thorough review of oral lymphangiomas reported that the mean age at the time of diagnosis was 22.7 years and that both sexes were equally affected.^4^ Most lymphatic malformations occur in the head and neck. In the oral cavity, such anomalies most commonly occur in the anterior two‐thirds of the tongue, but may also occur in the lips, gingiva, palate, and alveolar ridge of the mandible, whereas lymphatic malformation in the buccal mucosa is relatively infrequent.^1^, ^2^, ^3^, ^4^

The present case of lymphatic malformation in a 68‐year‐old woman is exceptionally unusual. Lymphatic malformation was not present at birth in our patient, but its development coincided with the patient regularly biting her buccal mucosa subsequent to a maxillary dental restoration. The etiology of acquired lymphatic malformation is unknown, but inflammation and trauma are thought to be likely causes. We found a total of five reported cases of lymphatic malformation of buccal mucosa by surgical treatment in an adult.^5^, ^6^, ^7^, ^8^, ^9^

There are several stages in the pathogenesis of vessel proliferation in acquired lymphatic malformation. First, the accumulation of interstitial fluid leads to swelling of the extracellular matrix, to which the endothelial cells lining the lymphatic capillaries become attached. Next, the endothelial cells lining the lymph ducts become elongated at the capillary level, β‐1‐integrin is activated, and phosphorylation of vascular endothelial growth factor receptor 3 occurs. This process results in the proliferation of endothelial cells in the lymph ducts, which is increased by edema and mechanical pressure.^10^, ^11^ It is thought that the inciting events in our patient were the edema caused by repeated biting of the buccal mucosa and the mechanical pressure exerted by chewing.

In our patient, the lesion involved the buccal mucosa but did not infiltrate the buccinator muscle. Therefore, it was possible to treat the patient surgically without damaging the parotid duct. Second, the lesion could be removed via the fascia of the buccinator muscle and the surgical wound completely closed by sutures.

When considering excision of a lymphatic malformation, five factors need to be taken into account, that is, the mortality risk, resectability of the malformation, functional effects, complications, and the likelihood of recurrence. In this case, the lesion was completely removed surgically without any residual dysfunction, such as trismus, or recurrence.

Spontaneous regression of a lymphatic malformation is rare, and various treatment strategies have been reported in the literature. In many reports, surgery or sclerotherapy was performed^12^, ^13^, ^14^ sometimes with laser therapy^15^ and less frequently with radiofrequency ablation.^16^ A case series of patients with lymphatic malformation reported that surgery and sclerotherapy were effective treatments. However, further studies are necessary to arrive at a consensus regarding optimal treatment. Treatment planning should consider the patient's age, type of pathology, anatomic location, growth rate, depth, dysfunction, and deformity.^17^ Although each of these factors is important, the type of pathology, anatomic location, and depth are the main determinants of the outcome.^1^

Lymphatic malformations are classified into three pathologic types, that is, macrocystic, microcystic, and mixed. The macrocystic type is usually lined by many layers and typically occurs in the neck, whereas the microcystic type has a single endothelial layer and typically occurs in the oral cavity. The lesion in our patient was of the typical microcystic type with an expanded vascular lumen that was stained by podoplanin. Sclerotherapy has often been reported to be of benefit in the treatment of lymphatic malformation of the microcystic type, albeit with wide differences in the rate of contraction of lesions.^18^, ^19^, ^20^, ^21^ Surgery is generally considered preferable to sclerotherapy if good results are expected, despite a lack of supportive evidence in the literature. Furthermore, there have been no well‐designed studies comparing sclerotherapy with surgery in the treatment of microcystic lymphangioma, which is understandable given the variations in the size and pathologic characteristics of these lesions.

Some authors do not recommend surgery as a first‐line treatment for lymphatic malformation because of relatively high rates of nerve injury, surgical site infection, and recurrence. Diffuse lesions have a high growth rate and may involve extensive amounts of surrounding tissue, which makes resection particularly difficult.^12^, ^13^, ^14^ However, surgery can be performed safely if these factors are ruled out. In terms of resection of the lesion, it is important that the range is superficial and does not involve the lymph ducts. If the lesion is completely removed surgically, the recovery time is shorter than for sclerotherapy. Therefore, we elected to perform surgery in our patient because the lesion was relatively small and involved only the interior of the buccinator muscle. However, we would be less keen to perform surgical resection in a patient with a lesion in the mucosa or a lesion that involved muscle.

## 4 CONCLUSION

Patient age, type of pathology, anatomic location, growth rate, depth, dysfunction, and deformity must be considered when planning treatment for a lymphatic malformation. When choosing surgery, the type of pathology, anatomic location, and depth are particularly important considerations. Acquired lymphatic malformation in an adult may proliferate in response to repeated irritation as a result of contact with the teeth. In our patient, the lesion was confined and did not involve adjacent vital structure. Surgery was chosen because the risk of complications was deemed to be minimal.

## CONFLICT OF INTEREST

None declared.

## AUTHORSHIP

AA: developed the concept and design of the study. KK: critically revised the manuscript for important intellectual content and gave the final approval of the version to be submitted. YI: drafted the article.
